# Nanoscale Band Gap Modulation and Dual Moiré Superlattices of Hexagonal Boron Nitride Weakly Coupled to Graphite

**DOI:** 10.1021/acsnano.5c09374

**Published:** 2025-10-02

**Authors:** Fábio J. R. Costa, Daniel Arribas, Thiago G. L. Brito, Tin S. Cheng, Jonathan Bradford, Amelia Thompson, Alex Saywell, Christopher J. Mellor, Peter H. Beton, Sergei V. Novikov, Juliette Plo, Bernard Gil, Guillaume Cassabois, Luiz Fernando Zagonel, Klaus Kuhnke, Klaus Kern, Anna Rosławska

**Affiliations:** † Gleb Wataghin Institute of Physics, University of Campinas − UNICAMP, Campinas 13083-859, Brazil; ‡ Max-Planck-Institut für Festkörperforschung, Heisenbergstraße 1, Stuttgart DE-70569, Germany; § School of Physics and Astronomy, 6123 University of Nottingham, Nottingham NG7 2RD, U.K.; ∥ Laboratoire Charles Coulomb, UMR5221 CNRS-Université de Montpellier, Montpellier 34095, France; ⊥ Institut Universitaire de France, Paris 75231, France; # Institut de Physique, École Polytechnique Fédérale de Lausanne, Lausanne CH-1015, Switzerland

**Keywords:** hexagonal boron nitride, moiré superlattices, van der Waals heterostructures, dual moiré, scanning tunneling microscopy, scanning tunneling spectroscopy

## Abstract

Van der Waals (vdW) materials, such as hexagonal boron nitride (h-BN), are highly promising for applications in optoelectronics and quantum technologies. When assembled into heterostructures, h-BN can form moiré superlattices, enabling the engineering of electronic and optical properties by varying the interlayer twist angle. However, understanding the nanoscale interplay between moiré patterns and electronic properties such as the band gap or work function, particularly in optically active h-BN structures, remains a challenge. Here, we use the atomic-scale precision of scanning tunneling microscopy (STM) to uncover the role of moiré superlattices in the electronic properties of a weakly coupled h-BN/Graphite heterostructure. Our STM study reveals large moiré patterns (14.8–18.3 nm periodicity) on the surface, implying slight local variations in the h-BN/Graphite stacking throughout the sample. Spectroscopic measurements show significant modulations of 330 meV in the local work function and 170 meV in the band gap within a moiré unit cell, which are comparable to h-BN/metallic interfaces. Additionally, we identify dual moiré superlattices in twisted homobilayers of h-BN/Graphite, offering an extra degree of freedom to tune the heterostructure’s properties. These findings suggest that moiré engineering in h-BN-based systems could lead to a range of effects, including exciton broadening, twist-tunable defect luminescence, and the theoretically predicted trapping of excitons within the moiré landscape. Furthermore, this tunability may also affect adjacent layered materials, providing a versatile platform for tailoring the electronic and optical properties of h-BN and its van der Waals heterostructures.

## Graphical abstract

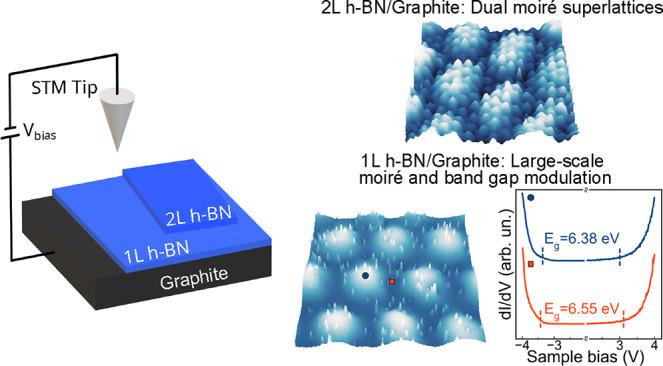

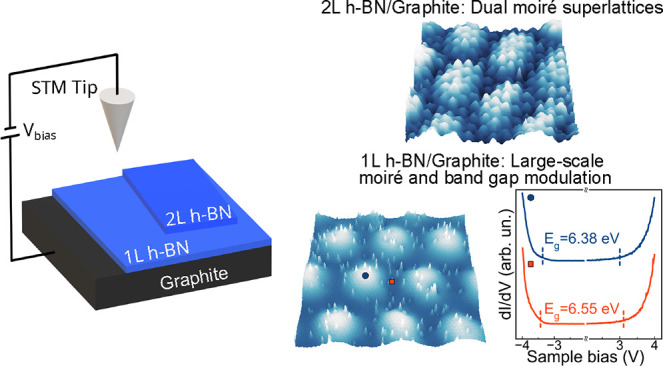

Atomically layered two-dimensional (2D) materials are excellent platforms for studying fundamental phenomena governing electronic transport and light–matter interactions, holding significant promise for applications in optoelectronics and nanophotonics.^1–5^ They are broadly categorized based on their band gap properties, with hexagonal boron nitride (h-BN) standing out as a versatile wide band gap/insulating material.^6,7^ As such, h-BN exhibits deep ultraviolet (DUV) luminescence in multilayer^8–10^ and single-layer^10–13^ thicknesses, also hosting luminescent point defects spanning the near-infrared to UV range.^14–19^ It also serves as an exceptional insulating and encapsulating layer in 2D heterostructures.^20–24^

Beyond their individual layers, different 2D systems can be used as building-blocks for van der Waals (vdW) heterostructures, where the stacking of atomically thin materials with differing properties often leads to exciting physics emerging at their interfaces.^25–29^ One prominent phenomenon is the formation of moiré superlattices (MSLs), periodic superstructures originating from lattice mismatch, and twist angles between adjacent layers. These structures enable nanoscale engineering of electronic potential landscapes, having driven breakthroughs such as unconventional superconductivity,^30^ the formation of secondary Dirac points in graphene,^31,32^ and moiré-trapped excitons in transition metal dichalcogenides (TMDCs).^33–38^ In h-BN-based moiré systems, twisting has been shown to modulate luminescence properties^39,40^ and to induce a ferroelectric behavior.^41,42^ Also, when composing vertical vdW heterostructures, twisted h-BN layers were shown to impede exciton diffusion in adjacent single-layered MoSe_2_,^43^ also forming moiré bands in neighboring TMDC layers via periodic dielectric screening.^44^

Understanding the localized effects of these MSLs remains a challenge, as many studies often employ diffraction-limited techniques that average out nanoscale modulations (≈10 nm) in the electronic structure. Therefore, addressing the interplay between moiré superlattices and local electronic properties requires higher spatial resolution, accessible via techniques such as scanning tunneling microscopy (STM), which offers subnanometer precision. While STM has been applied to explore moiré effects in graphene^45–47^ and TMDCs^48–50^ on weakly interacting substrates, such studies on weakly coupled h-BN systems remain scarce due to their insulating nature, which complicates STM measurements.

To date, the STM studies of moiré-induced electronic effects in h-BN have predominantly focused on samples grown on or transferred onto metallic substrates.^51–56^ While these investigations revealed localized modifications of electronic properties such as work function and band gap, the strong interlayer coupling with the metallic supports is known to quench the excitonic luminescence of atomically thin materials,^57–60^ limiting the possibility of correlating the electronic structure with the optical properties.

Considering these challenges, h-BN grown via plasma-assisted molecular-beam epitaxy (PA-MBE) on highly oriented pyrolytic graphite (HOPG) stands as an ideal system.^61–63^ This architecture combines conductivity for STM measurements with weak vdW interactions between the 2D layer and its support, maintaining h-BN’s intrinsic properties,^61,64^ which includes its UV excitonic luminescence.^11,58,65,66^ Previous STM characterization confirmed the preserved electronic band gap and successful luminescence excitation via current injection from the STM tip,^12^ though the extent of moiré-induced effects in this heterostructure remains underexplored.^12,66–68^

Motivated by this background, we investigate the role of moiré superlattices on the local electronic properties of h-BN/HOPG. Through STM imaging and spectroscopy, we reveal substantial moiré-induced modulations in work function and band gap. Moreover, studies on twisted h-BN bilayers reveal a dual MSL, a superposition of two independent superstructures, offering additional routes for twist-tuning the electronic properties of these heterostructures. Our results consolidate h-BN/HOPG as an optimal platform for exploring the importance of moiré superlattices for nanoscale studies, paving the way for future exploration of the interplay between these periodic potentials and the nanoscale optics in h-BN.

## Results and Discussion

### Moiré Superlattices in h-BN/HOPG

Figure 1 provides an overview of the sample analyzed in this study. STM measurements require the insulating h-BN layer to remain at a thickness of no more than a few monolayers. To meet this criterion, the growth conditions were optimized to favor moderate coverage, resulting in predominantly single-layer (1L) h-BN domains, as depicted in Figure 1a. Since both h-BN and HOPG share a hexagonal lattice with only a ∼2% bond-length difference (Figure 1b),^51,54^ atomically resolved images alone cannot reliably distinguish between these two isostructural surfaces. To address this limitation, scanning tunneling spectroscopy (STS) was employed to differentiate the two materials by their contrasting electronic properties, with h-BN being an insulator and HOPG a semimetal,^12^ as discussed in Supporting Figure S1.

**Figure 1:**
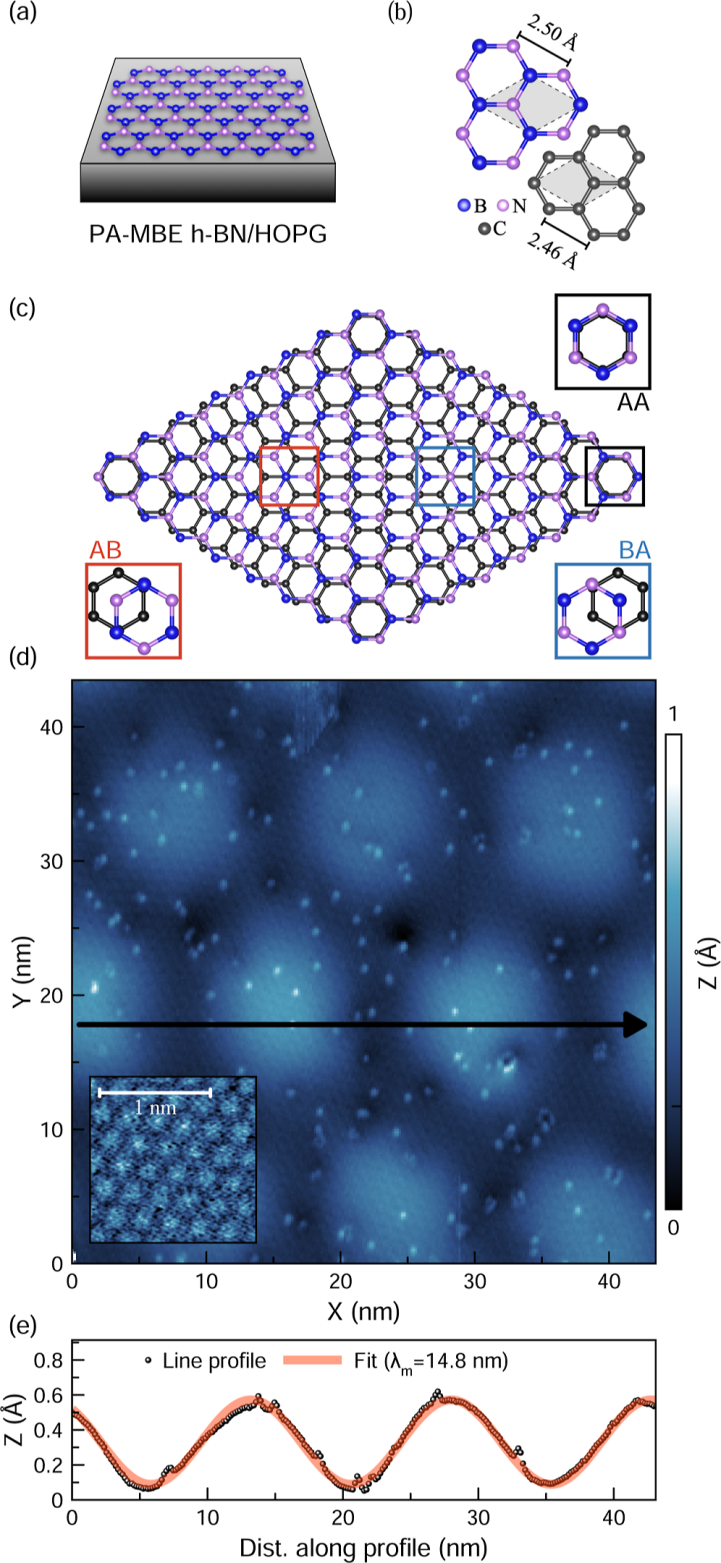
PA-MBE h-BN/HOPG. (a) Representation of the sample architecture. (b) h-BN and HOPG atomic lattices. The unit-cells are highlighted in gray, and the indicated distances correspond to the in-plane lattice constants. (c) Atomic scheme of the h-BN/HOPG moiré unit cell, highlighting different stacking configurations. The lattice mismatch was exaggerated to facilitate visualization. (d) Large-area scan of the h-BN/HOPG surface, acquired with a current set point of 2 pA and a sample bias of −3.5 V. The inset figure displays the atomically resolved h-BN lattice (2 pA, −3.5 V). (e) Line profile and fit along the arrow shown in (d).

The slight mismatch in lattice constants between h-BN and HOPG leads to the formation of MSLs, with their periodicity also influenced by the interlayer twist angle.^69^ Figure 1c illustrates the h-BN/HOPG interface, where the interaction between the two hexagonal lattices results in a gradual change in interlayer stacking configurations. Within a moiré unit cell, the atomic registry transitions gradually from AA stacking (B and N positioned atop C atoms) to AB/BA stacking (N/B located at the center of a C hexagon).^70^ Previous studies have shown that the adsorption sites of B and N at h-BN/metal interfaces significantly influence the system’s electronic properties, as the alignment between atoms at the boundary enhances hybridization and charge transfer effects.^51,52,55^ Therefore, similar registry variations within the nearly commensurate h-BN/HOPG stack are expected to induce comparable effects.

On a larger scale, the stacking variations in this vdW heterostructure can be perceived as extended moiré superstructures, exemplified by the region shown in Figure 1d, where periodic surface modulations in apparent height are observed. The elevated regions are referred to as “hills”, while the lower regions are “valleys”. In the same figure, an inset shows an atomically resolved image of the h-BN lattice from which a lattice constant of ∼0.26 nm was extracted. Figure 1e shows the line profile taken along the moiré, from which the superstructure’s dimensions can be estimated. Moreover, STM imaging revealed numerous nanometer-scale bright and dark point-like features on the surface, initially suggestive of defects in the h-BN epilayer. These sites appeared with significantly higher density in 1L h-BN compared to regions with thicker h-BN coverage (as shown in Figure 3 and further illustrated in Supporting Figure S2). A plausible interpretation is that these are plasma-induced defects in the HOPG surface, detectable through the atomically thin epilayer via STM.^71^ As h-BN becomes thicker, the additional insulating layers provide a sufficient barrier to suppress electrons tunneling into these defect states from HOPG, thus reducing their visibility in the STM, also accounting for the apparent lower defect density in thicker h-BN areas. Nonetheless, the observed features resemble a recent characterization of point defects in few-layer C-doped h-BN,^72^ and we cannot rule out the possibility of unintentional C-doping originating from the HOPG substrate.

Unless stated otherwise, all measurements presented in this study were performed on the same sample. Large moiré periodicity (λ_*m*_), as the case shown in Figure 1d,e, was consistently observed across different scanned regions located several micrometers apart. Supporting Figure S3 provides examples of additional areas and reveals moderate variations in periodicity across distinct regions. The observed superlattice dimensions ranged from 14.8 to 18.3 nm, implying that the h-BN/HOPG interface presents local variations within the same sample. Supporting Figure S4 shows the angular dependence of λ_*m*_ for the h-BN/HOPG heterostructure, from which a maximum value of ∼14 nm is predicted for an unstrained h-BN/HOPG interface.

The prevalence of superlattices whose dimensions were always above the aforementioned threshold for this heterostructure (λ_*m*_ > 14 nm) is an indicative of a slight compressive strain acting on the h-BN epilayer. Based on the observed moiré periodicities across the sample (λ_*m*_ between 14.8 and 18.3 nm), and also assuming no angular mismatch between h-BN and HOPG, we can estimate the compression of the epilayer’s lattice to be within the 0.17–0.49% range. This finding aligns with earlier AFM/STM studies on MBE-grown samples of both graphene/h-BN^68,73,74^ and h-BN/HOPG,^61,66–68^ which reported similar strain effects acting on the epilayers. The aforementioned variations observed between different scanning areas imply that the interaction between the h-BN epilayers and HOPG is not entirely uniform across the sample surface. In this context, it should be emphasized that both compressive and tensile strain are expected to alter the electronic structure of 1L h-BN, in particular by narrowing its band gap.^75,76^

### Moiré-Induced Effects in h-BN/HOPG

Periodic superstructures as the one shown in Figure 1d are a manifestation of the gradual changes in interlayer coupling along the dissimilar interface in a vdW heterostructure, resulting in a spatial modulation of the electronic properties of the system.^48,53,77^ These modulations have been related to two primary mechanisms: variations in interlayer spacing and changes in the interatomic registry. In the first case, the interlayer spacing (*d*) directly influences the electronic screening provided by the substrate, leading to band gap renormalization effects that scale approximately as 1/*d*.^78^ Notably, in constant-current STM measurements, the observed morphology reflects both electronic and topographical influences, making it challenging to quantify the interlayer corrugation from these measurements alone. In the current study, the measured height variations fall within the sub-Å range (Figure 1e), consistent with previous STM^52^ and AFM^56^ studies. Therefore, it is plausible that the apparent height modulation in the moiré could result in a relevant band gap renormalization within a moiré unit cell.

In parallel, the gradual shifts in atomic registry modulate the strength of interlayer interactions due to the highly directional nature of p_*z*_ orbitals in h-BN, resulting in regions of alternating strong and weak coupling.^51,55,77^ These combined effects create spatially periodic shifts in the electronic landscape, resulting in the spatial modulation of the work function and band gap in vdWHs.^48,51,53,55,56,61,77^ Notably, the magnitude of these modulations is also influenced by the dimensions of the MSL,^49,55,77^ with large superstructures (λ_*m*_ ∼10 nm) resulting in fluctuations in the range of several 100s of meVs.

To evaluate the extent of these moiré-induced effects in h-BN/HOPG, we conducted constant-current spectroscopy along the profile in Figure 2a, spanning the hills (H) and valleys (V) of the superstructure. The resulting spectra, shown in Figure 2b,c, reveal a series of field emission resonances (FERs). They are image potential states that experience a shift due to the strong electric field in the tip–sample junction and can be observed when the bias approaches the sample’s work function. These states can be understood as standing electron waves that form between the triangular potential barrier induced by the tip and the potential step at the surface, whose energies are related to the work function. While originally established in metallic systems and layered materials grown on conducting supports,^51,52,55,56^ similar FERs have been observed in graphite (semimetallic) and even in graphene grown on an insulating support,^79,80^ hence confirming their relevance in probing work function modulations in a broader range of sample configurations.

**Figure 2:**
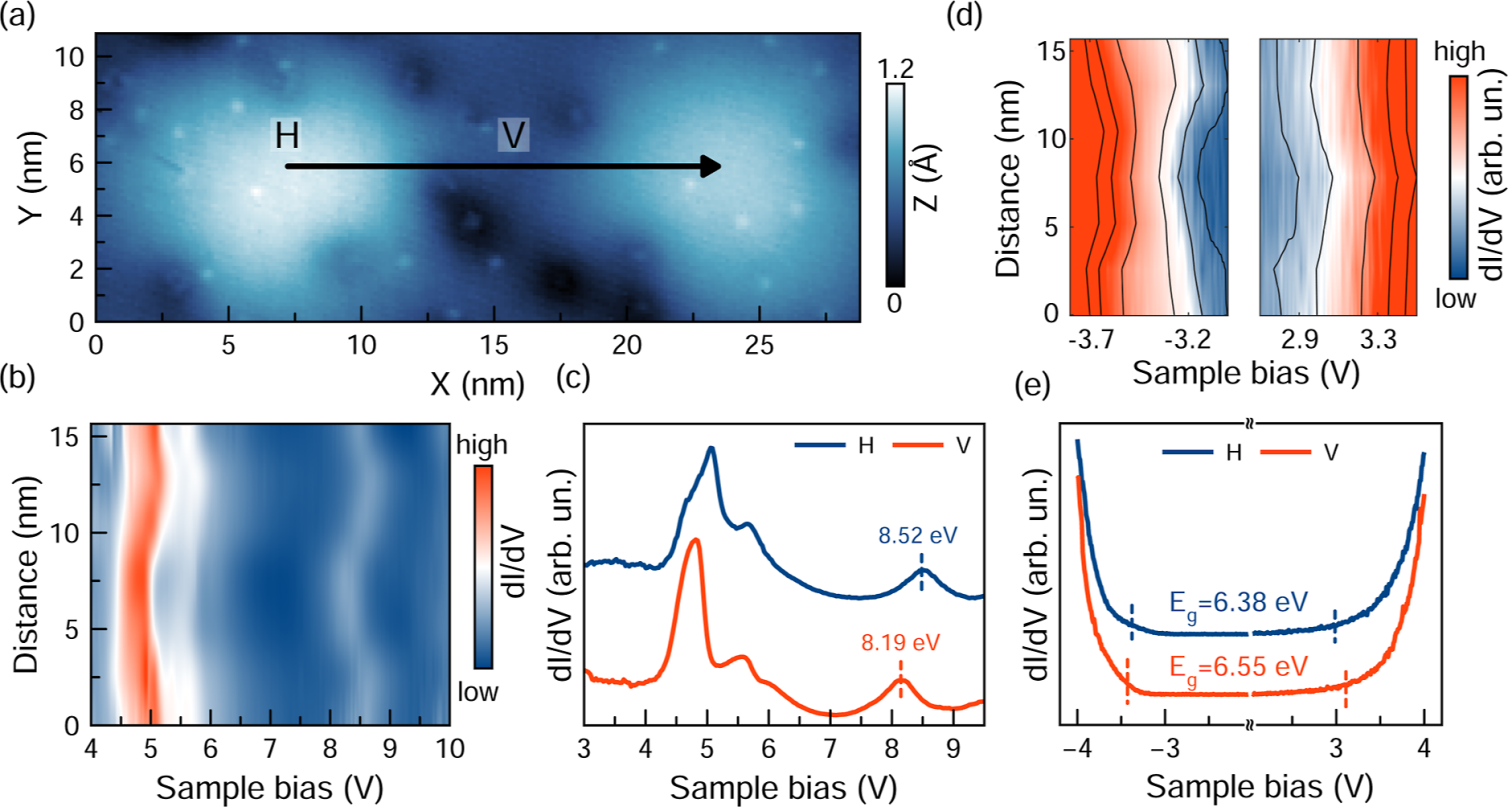
Moiré-induced electronic effects. (a) STM image of the moiré landscape (2 pA, −3.5 V). (b) Color-coded plot of constant-current dI/dV curves showing the spatial modulation of the FER peaks along the arrow indicated in (a). (c) Constant-current dI/dV spectra measured at the H and V positions of the moiré. Stabilization parameters: 100 mV, 1 nA. (d) Color-coded plot of constant-height dI/dV curves showing the spatial modulation of the valence and conduction band edges along the arrow indicated in (a). (e) Constant-height dI/dV curves measured at H and V of the moiré. Stabilization parameters: ±4 V, 50 pA. Spectra in (c) and (e) are normalized, and the blue curves are vertically shifted for clarity.

Based on these results, the shifting of the FERs over a moiré period in h-BN/HOPG can be used to evaluate the local change of the work function. Figure 2b shows the spatial modulation of the resonances along the line profile, and Figure 2c displays two spectra acquired at the H and V positions of the MSL. These constant-current measurements reveal two peaks, whose energies clearly depend on the tip position. The energy spacing between these resonances appears larger than what is observed in h-BN/metals,^51,52,55,56^ which can be assigned to a loss of tip sharpness^81^ as a consequence of scanning over nonconducting areas of the h-BN/HOPG sample in the initial large scans required to locate regions of interest. While the first-order resonances exhibit a slight energy shift, they also display a complex peak structure, as reported in previous studies and interpreted differently in the literature.^52,55,56^ Therefore, to achieve a more accurate evaluation, the second-order resonances (above 8 V) are used to quantify the moiré-induced work function shift. The dashed lines mark the energy of the local maxima in each spectrum, revealing a modulation of 330 meV on the work function between the H and V positions. As a comparison, moiré-induced modulations in the range of ∼100–400 meV were observed on h-BN samples grown on different metallic supports.^51–53,55,56^ Moreover, the moiré topography probed by STM imaging appears slightly inverted in the V regions when the imaging bias corresponds to the energies where the FERs occur, as further discussed in Supporting Figure S5.

One intriguing aspect is that, unlike h-BN/metal interfaces, where the presence of h-BN generally lowers the work function,^51,52^ with minima consistently observed at the sites of highest STM topography,^51,52,55,56^ our results on h-BN/HOPG reveal the opposite trend. The FERs are blue-shifted at H, and while previous studies on pristine HOPG^79^ and graphene/h-BN^80^ reported FER onsets around 4.2–4.3 V, our h-BN/HOPG spectra show onsets near 5.0 V, indicating an increase in work function upon h-BN deposition. These observations suggest that the interactions occurring at the h-BN/HOPG interface may lead to opposite trends compared to the h-BN/metal systems, even if the underlying mechanisms are similar in nature.

To probe the modulation of the band gap across the moiré unit cell, we performed a series of constant-height *dI*/*dV* measurements along the arrow indicated in Figure 2a. These measurements are sensitive to the local density of states of the surface, and in the context of a wide band gap material such as h-BN, the onsets in the *dI*/*dV* spectra at positive/negative bias can be associated with the conduction band minimum (CBM)/valence band maximum (VBM), respectively. The color-coded plots presented in Figure 2d show these onsets shifting along the profile, indicating substantial displacement of the band edges. To estimate the magnitude of such a modulation, individual *dI*/*dV* spectra are compared in Figure 2e, where the approximate band edge energies are indicated by dashed lines in the plot. See Supporting Figure S6 for more details on the assignment of the band edge energies. The comparison of measurements performed at the H and V positions of the MSL reveals a band gap modulation of 170 meV. Interestingly, this value is again comparable to the modulations observed for layered semiconductors adjacent to metallic supports, where a stronger interaction is expected. As examples, modulations of ∼130–150 meV were observed for TMDCs^53,77^ and ∼200–300 meV were reported on h-BN/Cu(111)^55^ and h-BN/Re(0001).^54^ Moreover, we note that tip–sample distances can influence spectroscopy measurements, and in Supporting Figure S7 we show that these effects act opposite to the moiré-induced modulations observed here, confirming that the band gap and work function variations arise from the h-BN/HOPG interaction rather than tip-height changes along the corrugated superstructure. The observation of electronic modulations of similar magnitude across a wide range of metallic supports, as well as for weakly interacting HOPG, seems puzzling at first. One might expect these effects to be more (less) pronounced in strongly (weakly) interacting interfaces. However, the absence of a clear trend suggests that the chemical nature of the supporting material plays a reduced role in these moiré-induced effects. Here, we distinguish between chemical interfacial interactions involving interlayer charge transfer, leading to possible chemical bonding with more reactive substrates, and electrostatic interfacial interactions (without significant interlayer charge transfer), which include weak vdW forces. In this context, X-ray photoelectron spectroscopy studies have shown clear evidence for charge transfer and chemical bonding between h-BN and metallic supports such as Re(0001)^54^ and Ru(0001),^82^ whereas for h-BN/HOPG, the same technique only detects C–C and B–N bonds,^61,64^ indicating a predominantly electrostatic interaction between the adjacent hexagonal materials.

Despite these different degrees of interfacial interactions, FER modulations on the strongly chemisorbed h-BN/Ru(0001)^54^ and on the weakly chemisorbed h-BN/Cu(111)^83^ fall within 130–150 meV.^53,56^ Similarly, band gap modulations in h-BN grown on weakly and strongly interacting supports (Cu(111)^55^ and Re(0001),^54^ respectively) remain near 200 meV. These values are comparable to our results (330 meV for FERs, 170 meV for the band gap), despite the weak vdW interaction between HOPG and h-BN. This suggests that the observed modulations arise primarily from the interplay of directional out-of-plane orbitals and registry-dependent variations within the moiré supercell, emphasizing that geometric and electrostatic effects can dominate over charge-transfer-based chemical interactions in determining these periodic electronic modulations.

In addition, the significant moiré-induced band gap modulation observed in h-BN/HOPG provides valuable insights into elusive aspects of previous studies on this PA-MBE-grown heterostructure. For instance, an earlier STM investigation^12^ reported a band gap of 6.8 ± 0.2 eV for single-layered h-BN/HOPG, with a broad uncertainty range attributed to a spatially varying interlayer coupling sampled across different areas with unresolved nanoscale topography. Our findings suggest that a substantial portion of this statistical fluctuation may instead arise from measurements taken at different positions along the moiré supercell, where such variations are intrinsic to the system. Moreover, a study investigating properties of excitons in monolayer h-BN/HOPG^65^ reported broad excitonic features in reflectivity spectroscopy. The inhomogeneous broadening (∼90 meV), probed using a large (∼100 μm)^11^ illumination spot, was attributed to spatial fluctuations in the interlayer coupling, which modify the local dielectric environment and, consequently, the excitonic response. In this context, our observation of band gap modulations within individual moiré unit cells further supports the interpretation that the dissimilar interface contributes to (or could be responsible for) the observed broadening. Since these band gap modulations can affect the material’s absorption and thus its reflectivity, averaging over many unit cells could naturally give rise to the inhomogeneous broadening observed in reflectivity data. Interestingly, the same study found that excitonic line widths probed via PL are much narrower, indicating a predominantly homogeneous broadening of the emission. This could suggest that while excitons in h-BN are sensitive to local band gap variations, they may diffuse and preferentially recombine at regions of reduced band gap within the moiré unit cell. Moreover, unlike conventional mechanically assembled heterostructures that are susceptible to contamination and disrupted interlayer coupling,^84–86^ our PA-MBE-grown h-BN/HOPG samples feature pristine interfaces. This ensures optimal moiré superstructure formation, allowing clearer observation of its effects and providing a robust platform for understanding these phenomena.

Finally, keeping in mind that the weak vdW coupling between h-BN and HOPG preserves the excitonic luminescence in this heterostructure,^11,12,65,66^ the observation of significant moiré-induced band gap modulations opens interesting opportunities to explore the interactions between these periodic superstructures and h-BN’s luminescence at the nanoscale. While this interplay between photoexcited excitons and twisted TMDC interfaces has been intensively investigated,^33–37^ similar phenomena in h-BN remain largely unexplored. Theoretical predictions suggest that twisted h-BN layers can form deep periodic potential wells capable of trapping excitons,^87^ while experimental studies have shown that stacking angles in multilayered h-BN can modulate the interface’s emission energy over ∼500 meV and enhance its intensity up to 40-fold.^39^ Also, defect-related luminescence could be twist-tuned in such a heterostructure to achieve a 12-fold photon count increase.^40^ Despite these advances, probing the interplay between MSLs and luminescence at the nanoscale in 2D materials remains challenging, and our results establish h-BN/HOPG as a viable platform for future investigations into the nano-optics of moiré-modulated vdW heterostructures.

### Dual Moiré Superlattices in 2L h-BN/HOPG

Even though the studied sample was synthesized aiming for predominant 1L h-BN coverage, one also expects the formation of some thicker h-BN islands. One of these uncommon regions is presented in Figure 3a. Scans over this area revealed a peculiar topography, where the left side presents the usual MSL, and on the right side, an additional h-BN layer exhibits its own MSL, superimposed on the underlying landscape.

**Figure 3:**
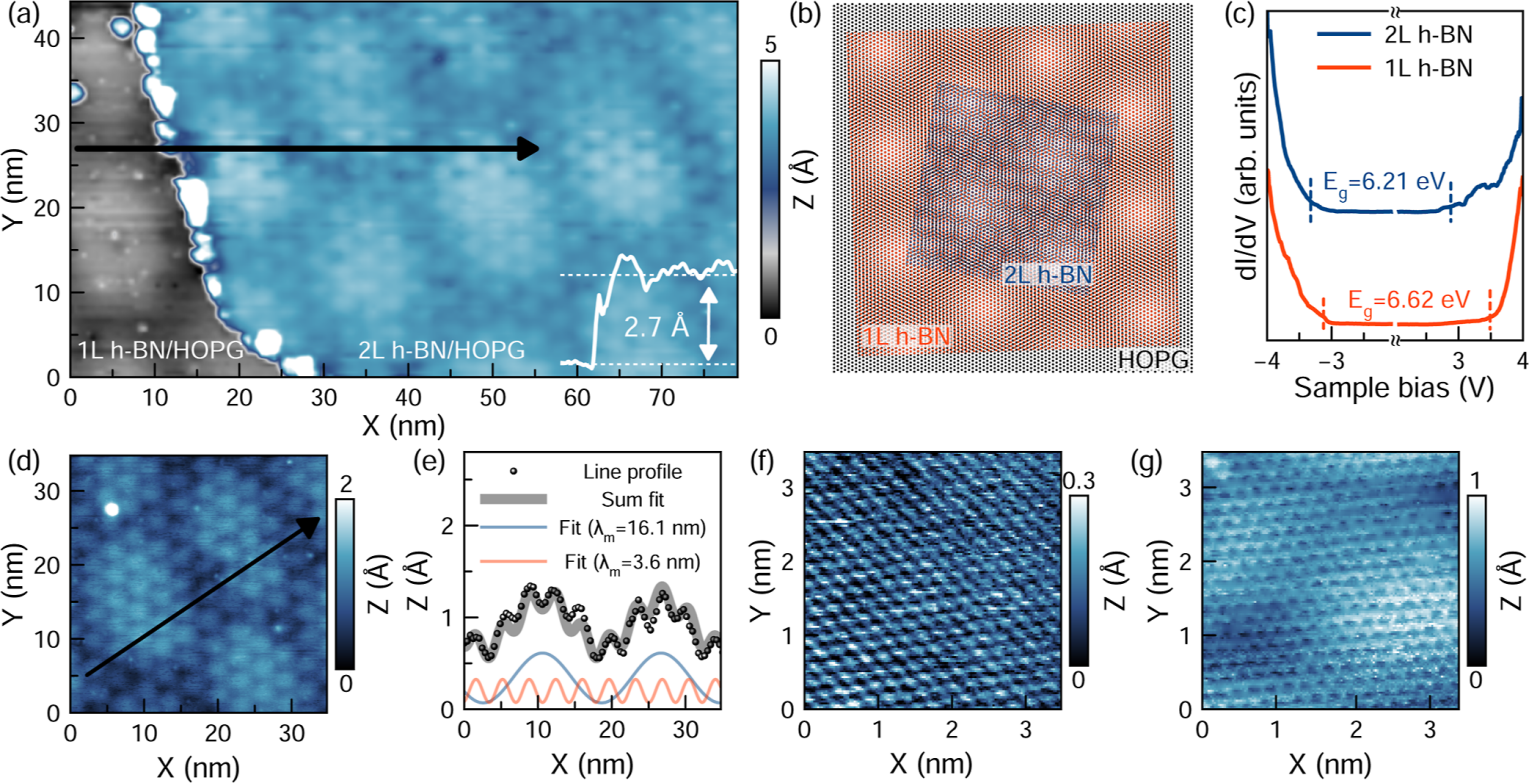
Dual moiré superlattice in 2L h-BN/HOPG. (a) Overview scan showing the edge of a second h-BN layer, with a second moiré superstructure superimposed on the moiré lattice of the structure below (2 pA, −3.5 V). The inset plot shows the step height between the h-BN layers, along the arrow. (b) Sketch of a 2L h-BN/HOPG, showing that the relative angle between h-BN layers results in a dual moiré pattern. (c) *dI*/*dV* spectra acquired on 1L and 2L h-BN/HOPG, showing a significant band gap reduction. Stabilization parameters: ±4 V, 40 pA. Spectra are vertically shifted for clarity. (d) Scan displaying the dual-moiré lattice in greater detail (3 pA, −3.5 V). (e) Line profile along the dual-moiré superlattice. Atomically resolved images acquired on 1L (f) and 2L (g) h-BN/HOPG (2 pA, −3.5 V).

This peculiar combination of moirés arises from the interference of two individual MSLs, in a 3-layered system with the appropriate twist alignments. Unlike the single MSL, whose periodicity is limited by the lattice constant mismatch between the stacked layers,^69^ the periodicity of the dual MSL is defined by the mismatch between the individual moirés, allowing one to tune the periodicity of the landscape even further.^88–90^ Such dual MSLs were previously reported in studies focusing on the h-BN/graphene/h-BN heterostructure, with a particular interest in the impacts of the complex landscape on the Dirac points in graphene’s electronic structure.^88–95^ Despite some earlier scanning probe studies,^43,96–98^ exploring the nanoscale geometry of these dual superstructures in real space requires nontrivial sample architectures, posing a considerable experimental challenge, and in particular, the consequences of these more complex superlattices on the intrinsic properties of semiconducting/insulating heterostructures remain underexplored.

An illustration of the dual MSL in a model trilayered system is presented in Figure 3b, where two misaligned layers of h-BN are placed over the HOPG lattice, producing an interference pattern analogous to the one observed in Figure 3a. To confirm the nature of the upper layer, we evaluated its thickness, based on the line profile shown as an inset in Figure 3a. The estimated height of 2.7 Å lies in the range reported in previous studies, where values of 2–4 Å were observed for the height of individual h-BN layers.^12,51,52,55,61,67^

Furthermore, the electronic structure of h-BN is sensitive to the thickness of the crystal, and a considerable band gap reduction is expected when going from one to two layers.^6,7^ To verify this effect, constant height *dI*/*dV* measurements were performed on both layers (Figure 3c), revealing a contraction of ∼400 meV in the band gap. Theoretical estimates for this effect show a significant variation between methods, with typical predictions lying within the 250–600 meV range,^6,7^ in line with the experimentally measured value. The spectra also show a sharper conduction band onset in the monolayer compared to the bilayer, which we attribute to differences in band dispersion:^6,7^ the nearly flat conduction band edge in 1L h-BN produces a rapid LDOS increase, whereas the more dispersed conduction states at the band edge in 2L h-BN lead to a less pronounced onset.

To quantify the periodicity of the overlying moiré lattice displayed in Figure 3d, we fit the profile to a sum of two sine functions yielding moiré periodicities of 3.6 and 16.1 nm. This confirms that the composite superstructure can be described as a superposition of the two independent MSLs. Considering the angular dependence displayed in Supporting Figure S4b, a moiré lattice with the aforementioned periodicity (λ_*m*_ = 3.6 nm) would require a mismatch in the range of 4° between two adjacent h-BN layers. This can be directly verified by comparing the lattice orientation on both levels of the structure. Atomically resolved images on both the 1- and 2-layered h-BN are displayed in Figure 3f,g, respectively. Both areas show a very similar hexagonal structure of ∼0.23 nm periodicity, where a slight misalignment between the lattices can be perceived. A quantitative assessment of this angular mismatch is presented in Supporting Figure S8, from which a relative rotation of (4 ± 0.7)° could be estimated. Moreover, other areas of multilayered h-BN exhibited a similarly consistent dual-moiré landscape, as detailed in Supporting Figures S9 and S10. Notably, recent studies have demonstrated that stacking-dependent electrostatic interactions at h-BN/h-BN^43^ and h-BN/graphene^99^ interfaces can induce periodic surface potential modulations. It is therefore reasonable to expect similar effects in our h-BN/h-BN/HOPG stack. However, the scarcity of suitable regions and challenging experimental conditions prevented a more detailed FER analysis in such areas. Nevertheless, in dual-moiré regions containing a domain boundary (Supporting Figure S10), we observe a pronounced bias-dependent contrast variation, consistent with distinct electronic structures across the boundary, highlighting that electronic properties can vary at the nanometer scale in this system.

## Conclusions

In our work, we investigated the structural and electronic effects of moiré superlattices in the weakly coupled h-BN/HOPG heterostructure. STM imaging revealed extensive moiré domains across the surface with periodicities ranging from 14.8 to 18.3 nm, suggesting slight local variations of the h-BN/HOPG interface along the PA-MBE-grown sample. Within a moiré unit cell, we observed the modulation of electronic properties, including a shift of 330 meV in the FERs, which indicates a work function modulation of similar magnitude. Additionally, we measured a moiré-induced bandgap modulation of 170 meV, comparable to values reported for h-BN on metallic substrates, where stronger substrate interactions are typically expected. This suggests that these modulations within a moiré unit cell are related to geometrical factors, implying that the chemical interaction with the support plays a minor role. Areas with twisted homobilayers of h-BN/HOPG were also characterized, revealing a dual moiré superlattice, with periodicities of 3.6 and 16.1 nm arising from the superposition of individual moirés. Furthermore, we observed a ∼400 meV band gap contraction between the single- and two-layered h-BN, consistent with theoretical predictions.

The observation of significant moiré-induced effects at the nanoscale paves the way for future investigations using the current h-BN/HOPG sample architecture. As a luminescent material, single-layered h-BN/HOPG supports intrinsic excitonic emission in the DUV, and the interaction between these excitons and the moiré superpotential is largely unexplored. Notably, a recent reflectivity study on h-BN/HOPG^65^ reported significant inhomogeneous broadening of the excitonic resonances, which may reflect moiré-induced effects across the heterostructure. Additionally, h-BN hosts a diverse range of optically active defects, which may also be influenced by the moiré landscape.^40^ Therefore, our findings are expected to inspire further research efforts combining STM and optical spectroscopies^12,58,84^ aimed at uncovering the nanoscale interplay between moiré superlattices and the rich luminescence properties of h-BN. In addition, the observed dual moiré superlattices offer an interesting opportunity to explore how these complex periodic potentials can be used to tailor the properties of multilayered heterostructures. Finally, given h-BN’s widespread use as a supporting/capping layer in various devices, the electronic structure modulations observed here could have important implications for the design and functionality of 2D-based devices.^5,36,43^

## Methods

### Sample Preparation

Monolayer h-BN was synthesized on an HOPG substrate by using PA-MBE. This method ensures the creation of single- and few-layered h-BN with atomically smooth surfaces and precise control over the sample thickness at the single-layer level. Control over the h-BN thickness and coverage is achieved by choosing the substrate temperature, the boron/nitrogen flux ratio, and the growth duration. For the specific sample under investigation, growth occurred at a sample temperature of approximately 1390 °C, utilizing a high-temperature effusion Knudsen cell for boron and a standard Veeco radio frequency plasma source for nitrogen. Further details regarding sample growth conditions and the MBE system are available elsewhere.^61–63,100^

### STM Imaging and Spectroscopy

We used a home-built STM setup operating in ultrahigh vacuum pressure (<10^–11^ mbar) and at low temperature (∼4.2 K). Prior to characterization, the h-BN/HOPG sample was annealed in ultrahigh vacuum for 4 h within the 500–650 K temperature range. All measurements utilized electrochemically etched Ag tips optimized via controlled indentations on a clean Ag(111) surface. The tip quality was confirmed by observing sharp atomic steps and the Ag(111) surface state in spectroscopy measurements.

STM imaging was carried out in a constant current mode. STS measurements were performed by using a lock-in amplifier with a bias modulation of 20 mV amplitude at a frequency of 691 Hz. In addition, constant-current spectroscopy measurements were performed, where the feedback remained active during the bias sweep, and the tip–sample distance was recorded. STM images were calibrated by using the h-BN atomic lattice as a reference. Based on this, we estimate a general uncertainty in distance measurements to be around 10%, attributed to image distortions caused by common artifacts such as piezoelectric creep and drift. Image analysis and processing were conducted using SPMImage Tycoon^101^ and Gwyddion,^102^ while spectroscopic data were analyzed using custom routines.

## Supplementary Material



## Notes

STM and STS data on an area of bare HOPG; comparison of defects in bare and h-BN-covered HOPG; details on the evaluation of the moiré dimensions and examples of additional areas showing large-scale superlattices; interlayer-angle dependence of the moiré periodicity for h-BN/HOPG and h-BN/h-BN interfaces; evaluation of the imaging contrast as a function of the applied bias; evaluation of the STS data; evaluation of the influence of tip–sample distance on the spectroscopy data; evaluation of the interlayer angle in a twisted bilayer of h-BN/HOPG; and additional observations of dual moiré superlattices in multilayered h-BN/HOPG (PDF)

## ¶

Université de Strasbourg, CNRS, IPCMS, 67034, Strasbourg, France
