# Supplementary material for: Nanoscale Band Gap Modulation and Dual Moiré Superlattices of Hexagonal Boron Nitride Weakly Coupled to Graphite
Source: ACS Nano. 2025 Oct 2;19(40):35528–38. doi: 10.1021/acsnano.5c09374 (PMC12530045; doi:10.1021/acsnano.5c09374)
Supplement: Supplementary file 1 [file nn5c09374_si_001.pdf]

Nanoscale Band Gap Modulation and Dual Moiré Superlattices of Hexagonal Boron Nitride Weakly Coupled to Graphite

Fábio J. R. Costa,[*,†,‡,#] Daniel Arribas,[‡] Thiago G. L. Brito,[‡] Tin S. Cheng,[¶] Jonathan Bradford,[¶] Amelia Thompson,[¶] Alex Saywell,[¶] Christopher J. Mellor,[¶] Peter H. Beton,[¶] Sergei V. Novikov,[¶] Juliette Plo,[§] Bernard Gil,[§] Guillaume Cassabois,[§,‖] Luiz Fernando Zagonel,[*,†] Klaus Kuhnke,[‡] Klaus Kern,[‡,⊥] and Anna Rosławska[*,‡]

†*Gleb Wataghin Institute of Physics, University of Campinas – UNICAMP, Campinas 13083-859, Brazil*

‡*Max-Planck-Institut für Festkörperforschung, Heisenbergstraße 1, DE-70569, Stuttgart, Germany*

¶*School of Physics and Astronomy, University of Nottingham, Nottingham, NG7 2RD, UK*

§*Laboratoire Charles Coulomb, UMR5221 CNRS-Université de Montpellier, 34095 Montpellier, France*

‖*Institut Universitaire de France, 75231 Paris, France*

⊥*Institut de Physique, École Polytechnique Fédérale de Lausanne, CH-1015 Lausanne, Switzerland*

#*Current address: Université de Strasbourg, CNRS, IPCMS, 67034, Strasbourg, France*

E-mail: fabio.costa@ipcms.unistra.fr; zagonel@unicamp.br; a.roslawska@fkf.mpg.de

Characterizing areas of uncovered HOPG

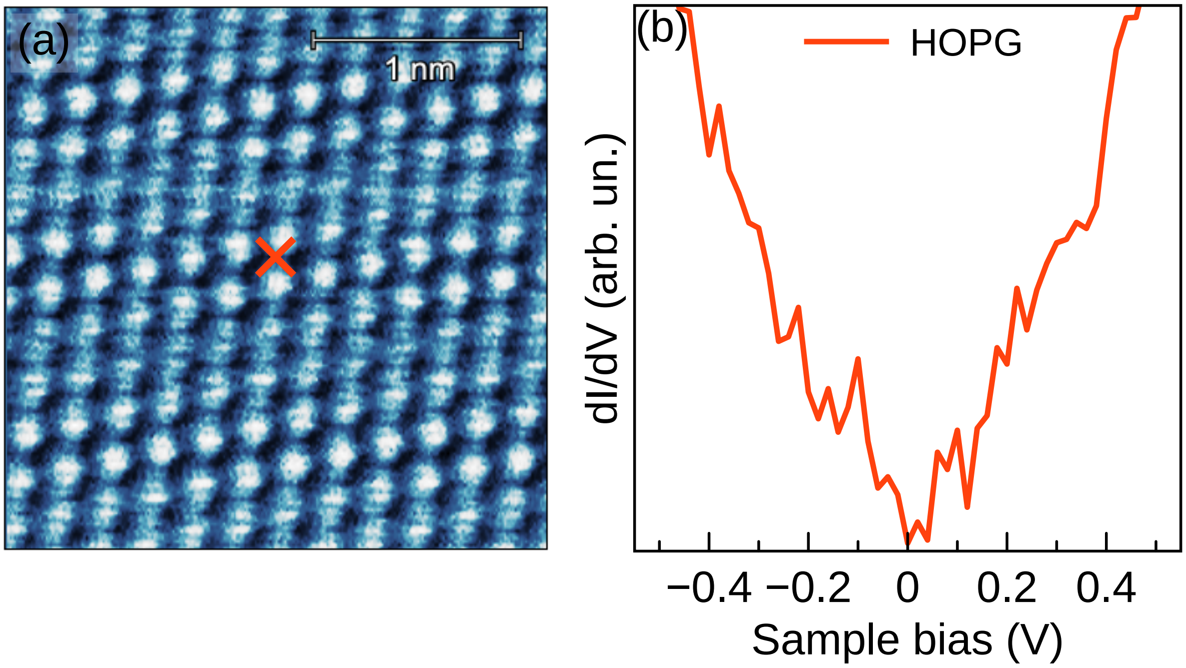

Figure S1: Characterization of an HOPG area. (a) Atomic lattice of HOPG, imaged at -0.1 V and 2 pA. (b) STS curve acquired on the red X in (a). Stabilization parameters: -1.1 V, 30 pA, with a lock-in modulation of 20 meV at 691 Hz.

Fig. S1(a) presents an atomically resolved STM image of the HOPG surface, revealing its hexagonal lattice with a periodicity of ∼0.24 nm. Given the potential influence of measurement artifacts, such as piezoelectric creep and drift, we estimate an error margin of approximately 10% when analyzing such images. Within this range, the 2% difference in lattice constants between h-BN and HOPG becomes negligible, making it difficult to distinguish between these isostructural surfaces relying solely on the STM-measured topography. However, this distinction can be achieved via STS measurements, as the two materials exhibit markedly different electronic properties: HOPG is semimetallic, while h-BN is insulating. Fig. S1 (b) displays a dI/dV spectrum acquired on HOPG, showing the characteristic linear dispersion around the Fermi energy expected for this material. In contrast, h-BN exhibits no differential conductance within its band gap bias range, as shown in Figs. 2(e) and 3(c) in the main text.

Comparison of defects in bare and h-BN-covered HOPG

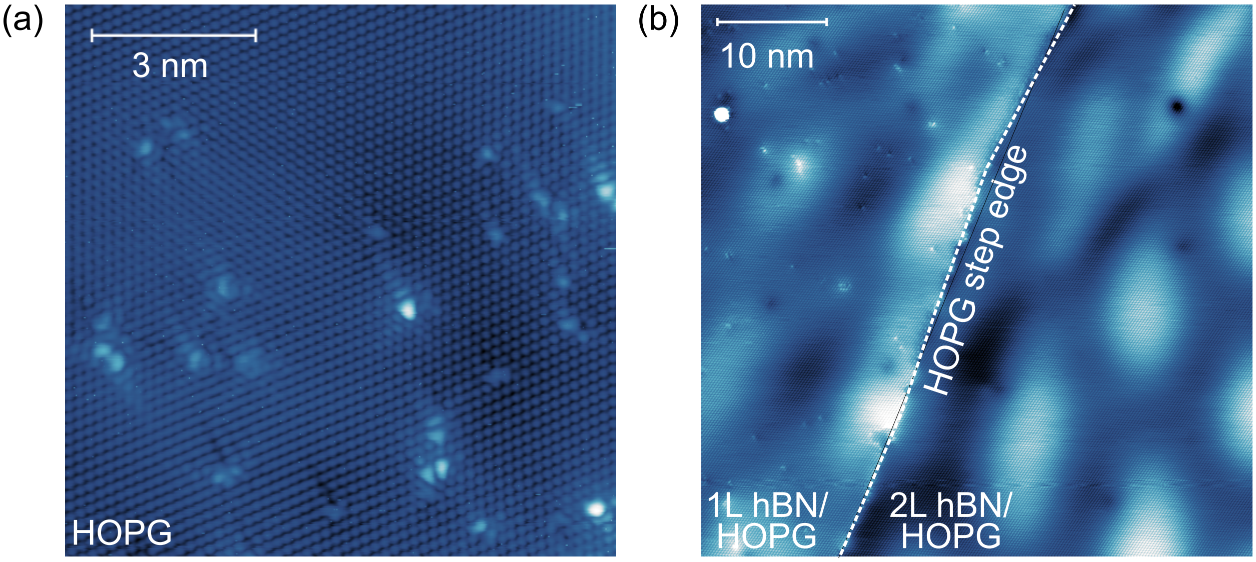

Figure S2: STM constant-current scans showing defects on the (a) HOPG surface (-3.7 V, 20 pA) and (b) at a boundary between 1L and 2L h-BN/HOPG (-3.6 V, 100 pA).

Figure S2 compares point-like features observed on bare HOPG (a) and in regions covered with 1–2 layers of h-BN (b). These measurements were performed on a different h-BN/HOPG sample, synthesized under similar conditions to the one shown in the main text. Data were acquired using a Scienta Omicron POLAR low-temperature STM, operated at 4.4 K and a base pressure of 10^{-10} mbar, with electrochemically etched W tips calibrated on a clean Au(111)/mica surface.

We interpret the high density of bright and dark features in 1L h-BN/HOPG regions (Fig. S2 (b)) as evidence of plasma-induced defects at the HOPG surface, formed during the h-BN growth. These features resemble those directly observed on exposed HOPG (Fig. S2 (a)), supporting the idea that they originate below the h-BN layer. The significantly lower defect density in thicker h-BN regions (right side of Fig. S2 (b)) suggests that additional layers can act as an electronic barrier, attenuating the tunneling current from buried defects and thus reducing their STM visibility in STM imaging. It is noteworthy that the right side of Fig. S2 (b) exhibits only a single moiré, which we assign to the lack of a small twist angle

between the two h-BN layers, contrasting the areas shown in Figs. 3, S9 and S10.

Moiré periodicity on different scanning areas

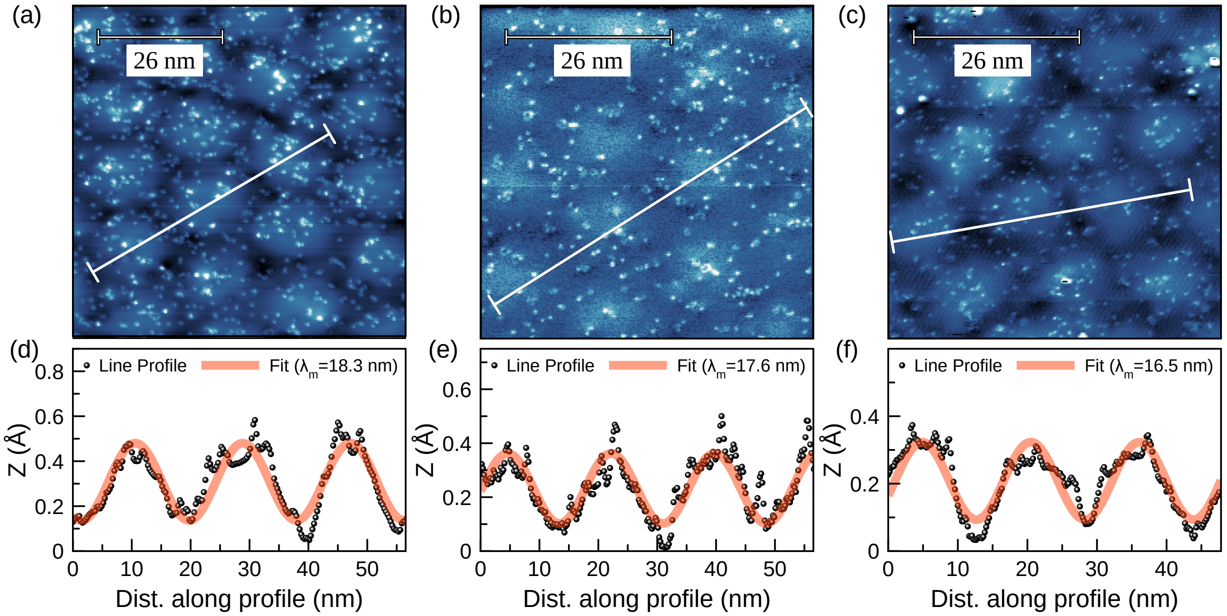

Figure S3: Comparison of moiré superlattices taken in different scanning areas across the sample surface (a-c) (2 pA, -3.5 V) and their respective line profiles (d-f).

Figures S3 (a-c) show large-area scans of moiré superlattices taken from different sample regions, spaced several μm apart. The superlattices exhibit some degree of asymmetry, which we attribute to typical piezo-related imaging distortions, such as creep and drift. Based on these effects, we estimate an uncertainty of approximately 10% in our measured distances. The observation of large-area moiré superlattices is consistent with previous studies on MBE-grown h-BN/HOPG, which reported moiré periodicities in the 14–18 nm range.[1–4] We extracted the moiré periodicity (λ_m) from our STM scans by fitting line profiles to sine functions, as shown in Figs. S3 (d-f). The measured range of λ_m (14.8–18.3 nm) suggests that the h-BN epilayer is under compressive strain, an interpretation further supported by Fig. S4, which shows the theoretical moiré periodicity as a function of the interlayer twist between h-BN and HOPG. For an aligned h-BN/HOPG interface, the expected maximum size is ~14 nm, whereas our experimental values consistently exceed this threshold. Producing these oversized moirés at a nearly aligned h-BN/HOPG heterostructure would require

a reduction of the interfacial lattice mismatch, which can be achieved by compressing the epilayer. To estimate the compressive strain leading to these enlarged superlattices, we assume perfect alignment (0º twist) between HOPG (lattice constant $a_{HOPG} = 0.246$ nm[5]) and h-BN, then use Eq. 1 to determine the h-BN epilayer's lattice constant in each area. The compressive strain is then computed as $\Delta a/a_{h-BN}$,[6] where Δa represents the deviation from h-BN's standard lattice constant. Based on this analysis, the observed moiré sizes correspond to a compressive strain of 0.17–0.49% acting in the h-BN epilayers.

Interplay between lattice mismatch and moiré periodicity

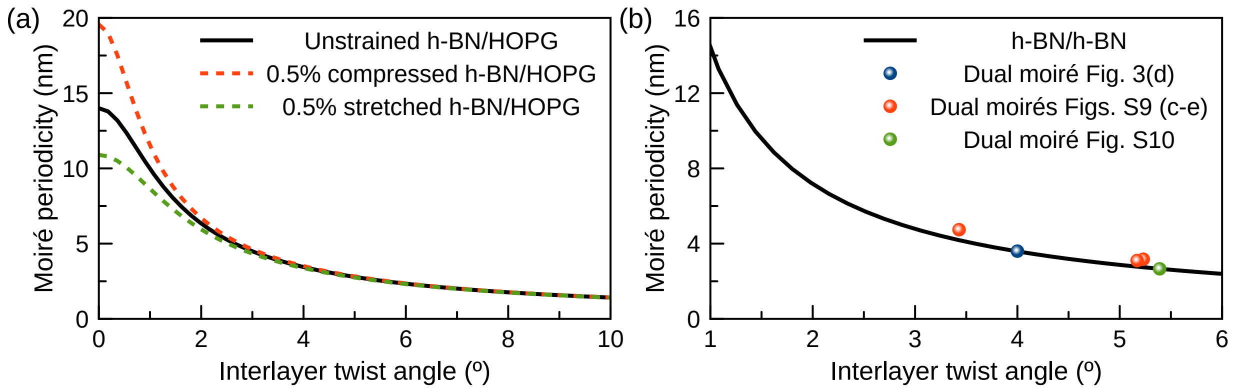

Figure S4: Moiré periodicities computed for h-BN/HOPG (a) and h-BN/h-BN (b).

Fig. S4 shows the angular dependence of moiré periodicity for h-BN/HOPG and h-BN/h-BN interfaces. Such estimations are based on the following equation:[7]

$$\lambda_m = \frac{(1+\delta)a_{\mathrm{i}}}{\sqrt{2(1+\delta)(1-\cos\theta)+\delta^2}} \tag{1}$$

Here θ is the relative angle between the adjacent layers, a_i=a_{HOPG} and $\delta = \frac{a_{\text{h-BN}} - a_{\mathrm{HOPG}}}{a_{\mathrm{HOPG}}}$ for the h-BN/HOPG heterostructure. For the h-BN/h-BN system, a_i=$a_{\text{h-BN}}$ and $\delta = 0$. For this analysis, we used the reference values a_{HOPG}=0.246 nm[5] and a_{h-BN}=0.250 nm.[5]

Bias-dependent imaging of moiré superlattices in h-BN/HOPG

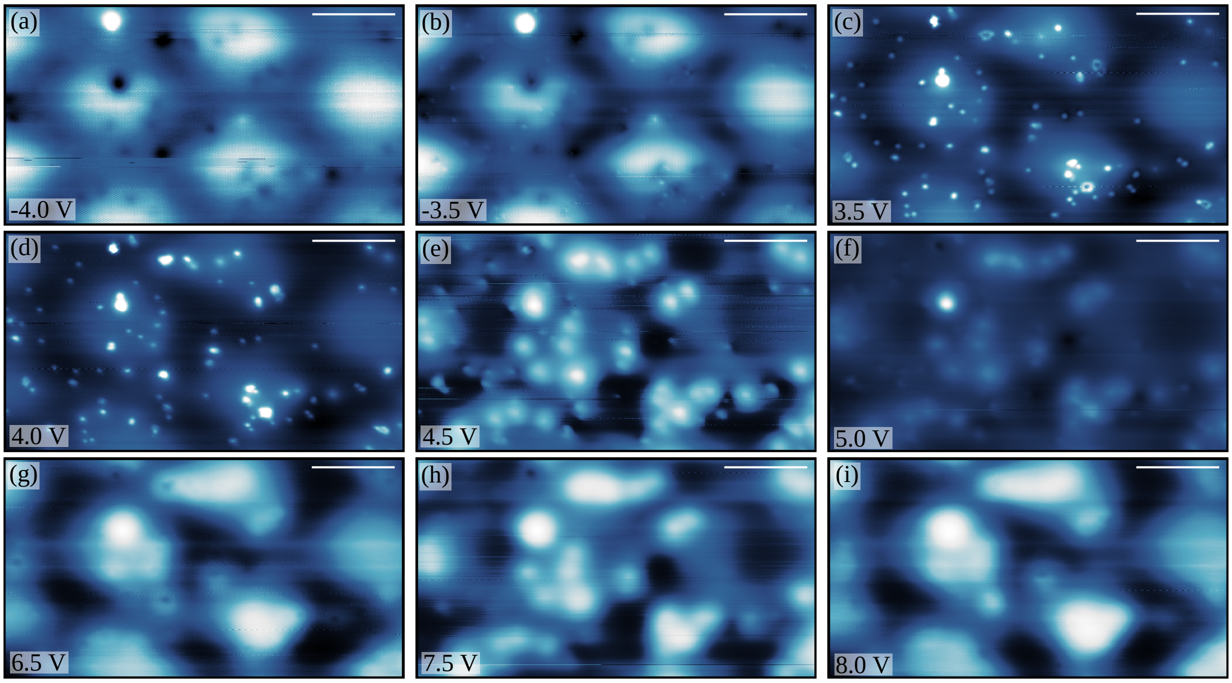

Figure S5: Bias-dependent imaging of h-BN/HOPG. Images were acquired at constant-current mode, with a 200 pA setpoint. The scale bars represent 10 nm.

Fig. S5 presents bias-dependent STM images of a moiré region in h-BN/HOPG, acquired between –4.0 V and +8.0 V. In the low-bias regime (–4.0 V to +4.0 V, Figs. S5 (a-d)), the contrast remains stable, with the hill (H) regions consistently appearing higher than the valley (V) regions. This behavior aligns with the band gap modulation observed in spectroscopy: H regions, having a reduced band gap, exhibit a higher LDOS, which in constant-current scans translates into a larger apparent height. At voltages corresponding to the first field-emission resonance (+4.5 V and +5.0 V, Figs. S5 (e,f)), the contrast becomes irregular, reflecting the complex spatial distribution of the resonances. The characteristic H–V contrast reappears at +6.5 V (Fig. S5 (g)). At +7.5 V (Fig. S5 (h)), however, the onset of the second FER in the V regions leads to a slight inversion of the H–V contrast, which is subsequently restored at +8.0 V (Fig. S5 (i)).

Estimation of band edges from STS data

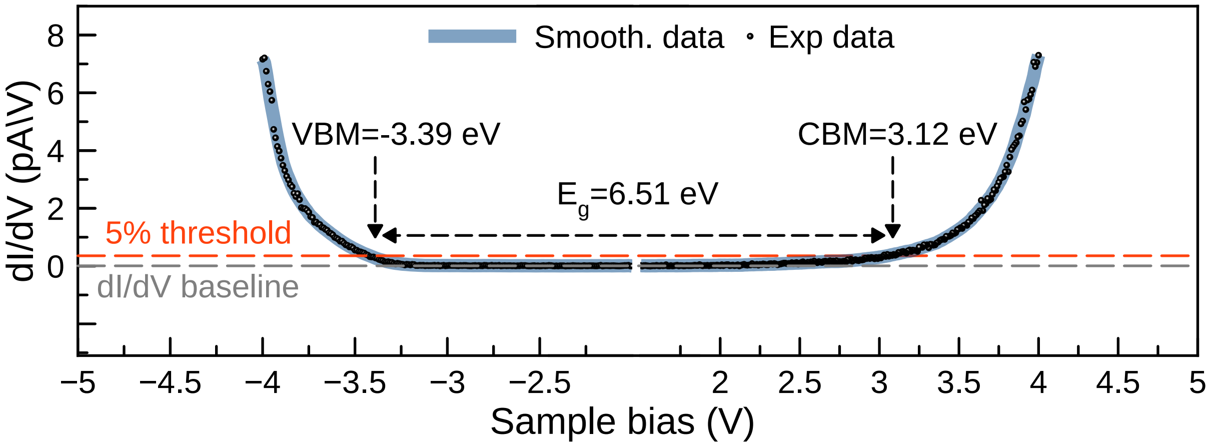

Figure S6: Determination of band-edges and baseline from STS curves.

The onsets of the conduction and valence bands (CBM and VBM, respectively) were determined from the STS data, following the approach illustrated in Fig. S6. To estimate these onsets, a baseline threshold is evaluated for each dI/dV curve, being computed as the average differential conductance within the +2.5 V to -3.0 V bias range, *a priori* assumed to comprise h-BN's band gap, where no relevant dI/dV signal above background was detected. As the sample bias was swept within the energies corresponding to h-BN's valence and conduction bands, the increasing local density of states led to a rise in dI/dV. Next, we evaluate the differential conductance range for each measurement, computed as the difference between the highest dI/dV reading and the baseline, and 5% of this range is taken as a threshold. Finally, the band onsets are estimated as the intersection between the smoothed STS curve and this 5% threshold above the baseline level.

Influence of tip-sample distance on the spectroscopy data

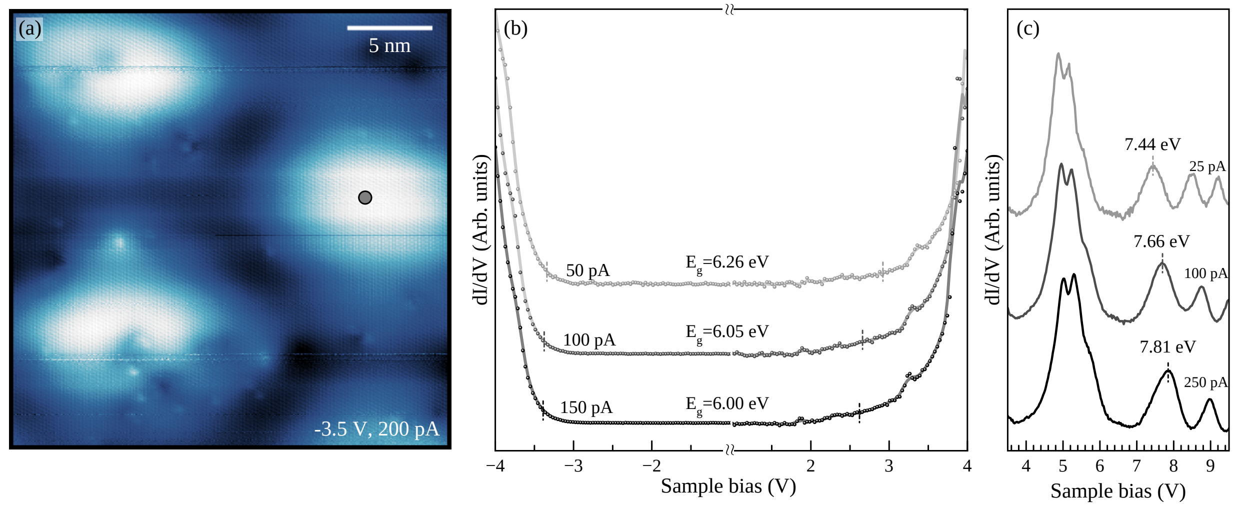

Figure S7: dI/dV spectra as a function of the tip-sample distance. (a) Overview scan of the h-BN/HOPG moiré. The gray circle marks the position where the measurements shown in (b) and (c) were taken. (b) Apparent band gap contraction as the tip-sample distance is reduced. Stabilization parameters: 50-150 pA, 4 V. (c) Apparent blue-shift of the field-emission resonances as the tip-sample distance is reduced. Stabilization parameters: 3.5 V, 25-250 pA. Spectra are vertically shifted for clarity.

As the tip–sample distance naturally varies across the moiré, spectroscopic measurements taken at different positions of the superlattice may start from different initial conditions. It is therefore essential to account for possible artifacts arising from these variations. To this end, we investigated the influence of the tip–sample distance by systematically changing the stabilization current setpoint (and also the tip-sample distance) before the bias sweep. The measurements were performed at the tip position marked by a gray circle in Fig. S7(a). The constant-height spectra (Fig. S7(b)) reveal a slight reduction of the apparent band gap as the stabilization current increases (tip–sample distance decreases), consistent with previous STM studies on h-BN.[8,9] This effect has been previously assigned to the variations on the signal to noise ratio induced by different setpoints, that may affect the detection of band onsets.[9] Most importantly, this behavior contrasts with the trend observed in Fig. 2, where the band gap increases when going from H (largest tip–sample distance) to V (smallest tip–sample distance). This confirms that the observed band gap variations cannot be attributed to

tip–sample distance variations along the moiré. Furthermore, the setpoint-dependent electric fields are also known to induce Stark shifts in the field-emission resonances,[10] and the current-dependent measurements (Fig. S7(c)) again follow an opposite trend to that in Fig. 2. Together, these findings rule out tip–sample distance effects as the origin of the measured work function and band gap modulations.

Evaluation of the interlayer alignment in 2L h-BN/HOPG

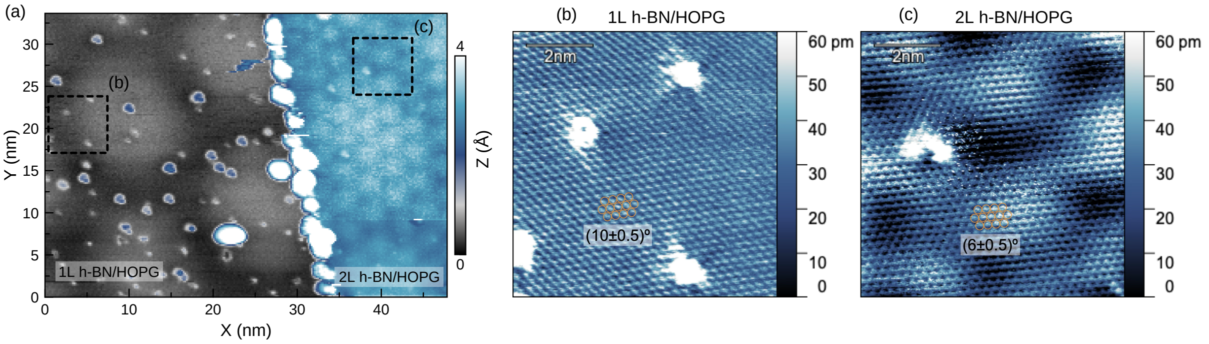

Figure S8: Relative angle between 1L and 2L h-BN/HOPG. (a) STM scan displaying the area of interest. The dashed squares highlight areas (b) and (c), that show the atomic arrangement of the lattice on 1L and 2L h-BN/HOPG, respectively.

Figs. S8(a-c), captured in the same area as Fig. 3 in the main text, provide atomically resolved images (Figs. S8(b-c)) that reveal the misalignment between the first and second h-BN layers. The orientation of atomic rows was measured relative to the scan direction, with an associated evaluation uncertainty of $\sigma = \pm 0.5°$. This yields an approximate interlayer angle of 4.0° with an uncertainty of $\sqrt{\sigma^2 + \sigma^2} = 0.7°$ between 1L and 2L h-BN/HOPG.

Additional observation of a dual moiré superlattice in multilayered h-BN/HOPG

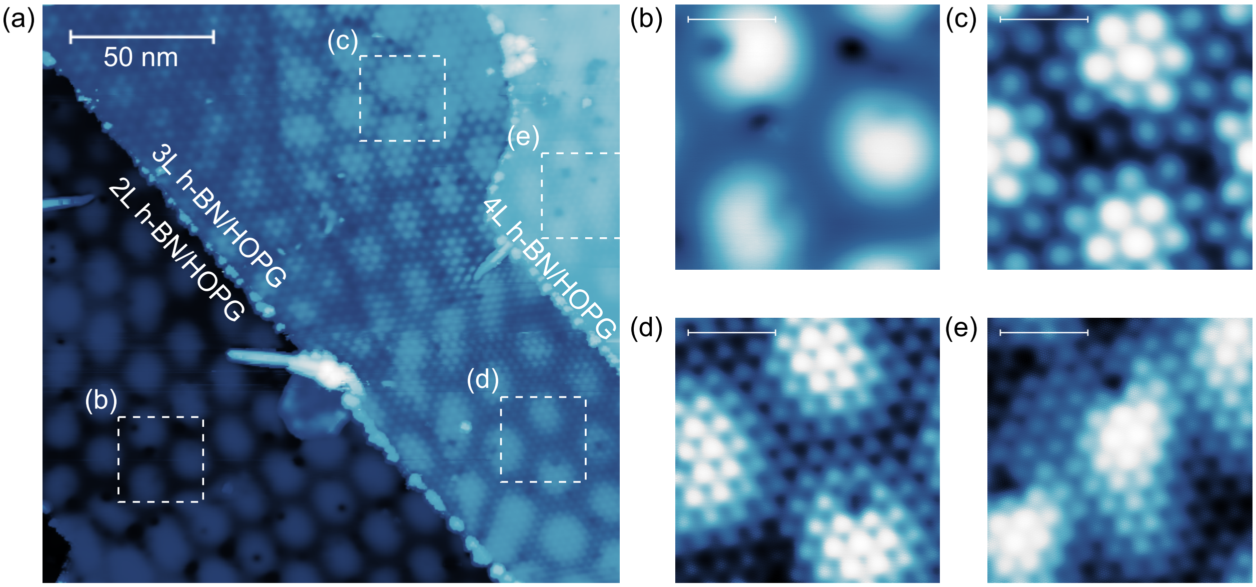

Figure S9: Dual moiré observed on another PA-MBE h-BN/HOPG sample. (a) Overview scan of an area exhibiting multi-layered h-BN thicknesses (-4 V, 20 pA). (b-e) Scans on the dashed areas highlighted in (a) (-3.6 V, 20-50 pA). The scale-bars shown in (b-e) correspond to 10 nm.

Figure S9 shows a large-area STM scan of a multilayer h-BN region, revealing a superposition of moiré patterns consistent with the observations in Fig. 3 in the main text. These measurements were performed on a different PA-MBE-grown h-BN/HOPG sample, using the same setup and experimental conditions as described for Fig. S2. Based on the low density of point defects, we tentatively assign the lowest terrace (left side of Fig. S9 (a)) to a 2L h-BN/HOPG stack with well-aligned h-BN layers, giving rise to a single moiré pattern with a periodicity of 19.7 nm. In contrast, the thicker regions corresponding to 3L and 4L h-BN (Figs. S9 (c–e)) exhibit dual moiré patterns, similar to those shown in Fig. 3. These additional moiré features have periodicities of 4.7, 3.2, and 3.1 nm, respectively. According to Eq. 1, these λ_m values correspond to relative rotation angles between h-BN layers in the range of 3.4°∼5.2°.

Electronic variations across dual-moiré boundaries

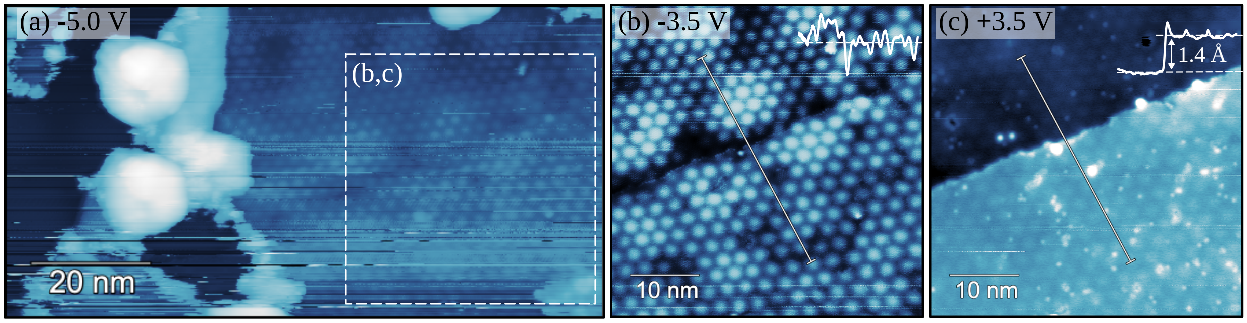

Figure S10: Additional measurements on a dual-moiré area. (a) Overview scan over an area showing coexisting domains exhibiting dual-moiré superlattices. (b-c) Closer scans showing the boundary between the two domains at different bias. The line profiles taken across the boundary are shown as insets. All images were acquired at 50 pA.

Fig. S10 presents additional measurements in a dual-moiré region of the same sample described in the main text. Thicker h-BN layers are typically found near nucleation sites (such as the white circular feature at mid-left side of Fig. S10(a)), where scanning is often unstable and prone to tip degradation. In this specific area, closer inspection of the dual moiré (Figs. S10(b–c)) reveals two coexisting domains. Interestingly, the boundary between these domains is barely discernible at –3.5 V, becoming clearly visible at +3.5 V, suggesting differences in their electronic properties.

References

(1) Cho, Y.-J.; Summerfield, A.; Davies, A.; Cheng, T. S.; Smith, E. F.; Mellor, C. J.; Khlobystov, A. N.; Foxon, C. T.; Eaves, L.; Beton, P. H.; Novikov, S. V. Hexagonal Boron Nitride Tunnel Barriers Grown on Graphite by High Temperature Molecular Beam Epitaxy. *Scientific Reports* **2016**, *6*, 34474.

(2) Summerfield, A.; Kozikov, A.; Cheng, T. S.; Davies, A.; Cho, Y.-J.; Khlobystov, A. N.; Mellor, C. J.; Foxon, C. T.; Watanabe, K.; Taniguchi, T.; Eaves, L.; Novoselov, K. S.; Novikov, S. V.; Beton, P. H. Moiré-Modulated Conductance of Hexagonal Boron Nitride Tunnel Barriers. *Nano Letters* **2018**, *18*, 4241–4246.

(3) Thomas, J.; Bradford, J.; Cheng, T. S.; Summerfield, A.; Wrigley, J.; Mellor, C. J.; Khlobystov, A. N.; Foxon, C. T.; Eaves, L.; Novikov, S. V.; Beton, P. H. Step-flow growth of graphene-boron nitride lateral heterostructures by molecular beam epitaxy. *2D Materials* **2020**, *7*, 035014.

(4) Wang, P. et al. Scalable Synthesis of Monolayer Hexagonal Boron Nitride on Graphene with Giant Bandgap Renormalization. *Advanced Materials* **2022**, 2201387.

(5) Qi, Y.; Han, N.; Li, Y.; Zhang, Z.; Zhou, X.; Deng, B.; Li, Q.; Liu, M.; Zhao, J.; Liu, Z.; Zhang, Y. Strong Adlayer–Substrate Interactions "Break" the Patching Growth of h-BN onto Graphene on Re(0001). *ACS Nano* **2017**, *11*, 1807–1815.

(6) Summerfield, A.; Davies, A.; Cheng, T. S.; Korolkov, V. V.; Cho, Y.; Mellor, C. J.; Foxon, C. T.; Khlobystov, A. N.; Watanabe, K.; Taniguchi, T.; Eaves, L.; Novikov, S. V.; Beton, P. H. Strain-Engineered Graphene Grown on Hexagonal Boron Nitride by Molecular Beam Epitaxy. *Scientific Reports* **2016**, *6*, 22440.

(7) Yankowitz, M.; Xue, J.; Cormode, D.; Sanchez-Yamagishi, J. D.; Watanabe, K.; Taniguchi, T.; Jarillo-Herrero, P.; Jacquod, P.; LeRoy, B. J. Emergence of superlat-

tice Dirac points in graphene on hexagonal boron nitride. *Nature Physics* **2012**, *8*, 382–386.

(8) Román, R. J. P.; Costa, F. J. R. C.; Zobelli, A.; Elias, C.; Valvin, P.; Cassabois, G.; Gil, B.; Summerfield, A.; Cheng, T. S.; Mellor, C. J.; Beton, P. H.; Novikov, S. V.; Zagonel, L. F. Band gap measurements of monolayer h-BN and insights into carbon-related point defects. *2D Materials* **2021**, *8*, 044001.

(9) Qiu, Z.; Vaklinova, K.; Huang, P.; Grzeszczyk, M.; Watanabe, K.; Taniguchi, T.; Novoselov, K. S.; Lu, J.; Koperski, M. Atomic and Electronic Structure of Defects in hBN: Enhancing Single-Defect Functionalities. *ACS Nano* **2024**, *18*, 24035–24043.

(10) Su, W. B.; Lu, S. M.; Jiang, C. L.; Shih, H. T.; Chang, C. S.; Tsong, T. T. Stark shift of transmission resonance in scanning tunneling spectroscopy. *Physical Review B* **2006**, *74*, 155330.
